# Harnessing natural terpenes via PEGylated mucoadhesive vesicles for improved urinary bladder delivery: from *in vitro* evaluation to *in vivo* assessment

**DOI:** 10.3389/fphar.2025.1685423

**Published:** 2025-12-18

**Authors:** Hayder A. Hammoodi, Rofida Albash, Mohammed I. A. Hamed, Khaled M. Darwish, Amira B. Kassem, Asmaa Saleh, Jawaher Abdullah Alamoudi, Noha M. Badawi

**Affiliations:** 1 Department of Pharmacy, Mazaya University College, Thi-Qar, Iraq; 2 Department of Pharmaceutics, College of Pharmaceutical Sciences and Drug Manufacturing, Misr University for Science and Technology, Giza, Egypt; 3 Department of Organic and Medicinal Chemistry, Faculty of Pharmacy, Fayoum University, Fayoum, Egypt; 4 Department of Medicinal Chemistry, Faculty of Pharmacy, Galala University, New Galala, Egypt; 5 Department of Medicinal Chemistry, Faculty of Pharmacy, Suez Canal University, Ismailia, Egypt; 6 Department of Clinical Pharmacy and Pharmacy Practice, Damnhour University, Damnhour, Egypt; 7 Department of Pharmaceutical Sciences, College of Pharmacy, Princess Nourah Bint Abdulrahman University, Riyadh, Saudi Arabia; 8 Department of Pharmaceutics and Pharmaceutical Technology, Faculty of Pharmacy, The British University in Egypt, Cairo, Egypt

**Keywords:** intravesical delivery, fenticonazole nitrate, terpesomes, chitosan, gelucire, confocal laser scanning microscopy, histopathological study

## Abstract

**Background:**

Fenticonazole nitrate (FTN)-loaded chitosan-coated PEGylated terpesomes (TPs) were investigated as a mucoadhesive formulation to enhance drug delivery to the urinary bladder.

**Methods:**

The design of fenticonazole nitrate-loaded terpesome (FTN-TP) formulations was carried out using a 3^1^.2^1^ factorial design. The vesicles were evaluated for entrapment efficiency (EE%), particle size (PS), polydispersity index (PDI), and zeta potential (ZP). The selected FTN-TPs were further mixed with Gelucire^®^ 44/14 as a PEG source and coated with chitosan to obtain the optimized chitosan-coated PEGylated terpesomes (CCPTs), which were then subjected to additional characterization.

**Results:**

The optimized CCPTs exhibited increased entrapment efficiency and zeta potential values without a significant increase in particle size or polydispersity index. They also demonstrated good mucoadhesive properties and remained stable for up to 90 days. Confocal laser scanning microscopy confirmed the penetration of the fluorescently optimized CCPTs through the urinary bladder wall. *In silico* studies indicated good stability of FTN when combined with other components in the optimized CCPTs. Moreover, *in vivo* evaluation revealed a significantly greater antifungal effect in rats treated with the optimized CCPTs compared to those receiving the FTN suspension. Histopathological examination further confirmed the safety of the optimum formulation for urinary bladder administration.

**Conclusion:**

The optimum formulation could serve as an effective alternative carrier for urinary bladder delivery of fenticonazole nitrate, prolonging its residence time and thereby enhancing its therapeutic pharmacological effect.

## Graphical Abstract

**Figure:**
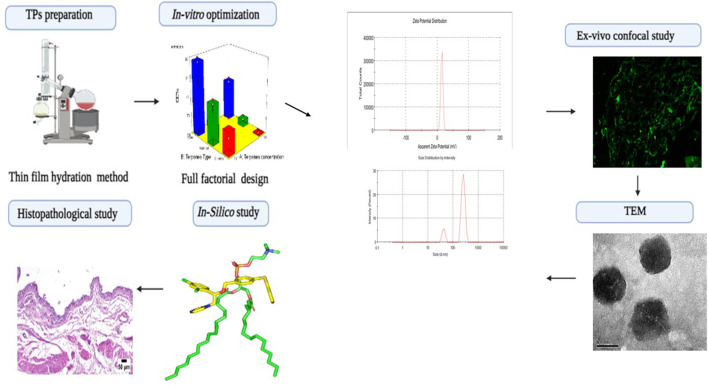

## 1 Introduction

Intravesical drug delivery involves inserting a urethral catheter into the bladder to administer medications directly. This route is frequently used to treat bladder-related disorders such as interstitial cystitis and bladder cancer (Kolawole et al., 2017; Mackie et al., 2017). It offers the advantage of reducing systemic side effects while increasing the amount of drug that reaches the target tissues. In contrast, oral administration is often unsuitable for bladder diseases because renal excretion, metabolism, and limited absorption reduce drug bioavailability (Kaldybekov et al., 2018). Despite its benefits, intravesical delivery has several drawbacks, including the need for intermittent catheterization, low urothelial permeability, and significant drug dilution and washout. The procedure may also cause discomfort, and in some cases, infections or inflammatory reactions could occur (GuhaSarkar and Banerjee, 2010).

Mucoadhesive formulations have been introduced to address these challenges by improving drug residence and permeability within the bladder (Andrews et al., 2009). Mucoadhesive materials can attach to the bladder epithelium and resist urine washout, thereby extending drug contact time and enhancing bioavailability (Kaldybekov et al., 2019). To be effective, such systems must not disturb normal bladder physiology and should maintain strong adhesion for several hours, even after urination. The dosage form should also adhere rapidly and firmly to the bladder mucosa (Kaldybekov et al., 2018).

Both synthetic and natural hydrophilic polymers have been explored for their mucoadhesive potential, including chitosan, carbomers, and cellulose derivatives (Khutoryanskiy, 2011; Kaldybekov et al., 2018). Their adhesion resulted from interactions with mucin, such as hydrogen bonding, electrostatic attraction, diffusion, and chain entanglement (Davidovich-Pinhas and Bianco-Pele, 2014). In comparative studies, chitosan exhibited stronger adhesion to porcine bladder mucosa than carboxymethylcellulose, leading to slower drug release (Burjak et al., 2001).

Polyethylene glycol (PEG) is extensively utilized in biomedical applications (D’souza and Shegokar, 2016). Its inclusion in formulations enhances bonding between polymers and the urothelial mucosa (Sarfraz et al., 2022). Moreover, PEGylation can create covalent bonds with thiol groups in the mucin of the bladder, improving mucoadhesion (Kaldybekov et al., 2018).

Nanotechnology offers additional advantages through carriers in the nanometer size range (Balaji et al., 2025). These nanocarriers can be delivered into the bladder via a catheter to achieve localized therapy. A variety of nanomaterials have been tested for intravesical drug delivery (Globig and Hunter Jr, 2011; Sarfraz et al., 2022). Among these, terpesomes (TPs), vesicular systems containing terpenes, have shown considerable promise. They were first introduced by Albash et al. (2021a) for the treatment of ocular candidiasis.

Terpenes are natural organic metabolites composed of repeating isoprene units and are found in essential oils (Tetali, 2019). In addition to their well-known penetration-enhancing properties, terpenes exhibit antimicrobial and antifungal effects due to their ability to interact with lipid membranes, leading to cell damage (Teaima et al., 2022). Mucoadhesive TPs have also been formulated for vaginal delivery of propranolol hydrochloride using various terpenes and chitosan (Teaima et al., 2022). Chitosan plays a dual role as both an antifungal agent, by disrupting fungal cell membranes, and as a mucoadhesive coating material (Eltabeeb et al., 2024).

Cystitis is an inflammatory disorder of the bladder that may be caused by bacterial or fungal infection, but it can also occur without infection, as in bladder pain syndrome or interstitial cystitis (Ismail and Hashim, 2013). Fungal cystitis is diagnosed by identifying hyphae on urine microscopy, culture and sensitivity tests, or histopathological examination (Guarner and Brandt, 2011). A fungal count exceeding 10^3^ CFU/μL in urine indicates funguria (Etienne and Caron, 2007). Treatment is guided by the severity of symptoms and the patient’s general condition (Fisher et al., 2011).

Azoles are a class of antifungal agents characterized by the imidazole ring. Fenticonazole nitrate (FTN) inhibits ergosterol synthesis, thereby disrupting fungal cell membranes. It exhibits both fungicidal and fungistatic activity (Albash et al., 2021b). However, its poor aqueous solubility (<0.10 mg/mL) necessitates the development of a more efficient vesicular delivery system (Albash et al., 2022).

FTN has previously been formulated as novasomes and olaminosomes to improve ocular permeability and enhance antifungal activity (Ahmed et al., 2022a). It was also incorporated into limonene-enriched cubosomal dispersions to extend corneal residence time and improve bioavailability (Nemr et al., 2024). To date, no study has investigated the encapsulation of FTN in vesicles modified with both a penetration enhancer and a mucoadhesive polymer for intravesical application.

Accordingly, the present work describes the formulation, optimization, and characterization of chitosan-coated PEGylated terpesomes (CCPTs) loaded with FTN as a model intravesical delivery system. This study aimed to enhance the intravesical distribution of FTN and evaluate its safety using a 3^1^.2^1^ factorial design. The selected formulation was coated with chitosan to impart a positive charge and incorporated a PEG-containing surfactant (Gelucire 44/14) to enhance mucoadhesion. The optimum formula underwent confocal laser scanning microscopy, mucoadhesion testing, and a 3-month stability study, followed by molecular docking and dynamics simulation. Finally, *in vivo* evaluation compared the antifungal pharmacological effect of the optimized CCPTs with FTN suspension, and histopathological analysis was performed to assess safety.

## 2 Materials and methods

### 2.1 Materials

FTN was provided as a gift by Andalous Pharmaceutical (Cairo, Egypt). Phospholipid (PC) from egg yolk, mucin, and chitosan [LMW 100–300 kDa] were purchased from Sigma-Aldrich (St. Louis, United States). Cineole, citral, and menthol were purchased from Alfa Aesar (Germany). Methanol (HPLC grade) was obtained from El-Nasr Pharmaceutical Co. (Cairo, Egypt). Gelucire (44/14) was obtained from Gattefosse (Cedex, France).

### 2.2 Preparation of terpesomes

TPs were prepared using cineole, citral, and menthol, each incorporated at concentrations of 10 or 30 mg, following thin-film hydration (Table 1). Precisely weighed quantities of PC (100 mg), terpene, and FTN (10 mg) were dissolved together in methanol within a round-flask. The solvent was evaporated under reduced pressure at 60 °C using a rotary evaporator (Heidolph, Germany) with a rotation speed of 90 rpm, resulting in the formation of a thin lipid film. This film was subsequently hydrated with 10 mL of aqueous phase at 60 °C to yield vesicular dispersion (Teaima et al., 2022). Furthermore, beads were added during the hydration to facilitate the complete dispersion of the film. The resulting terpesomal dispersion was kept at 4 °C until the final formulations were obtained.

**TABLE 1:** Full factorial design for FTN-TPs.

Factor	Levels	
X_1_: Terpene type	Cineole menthol	Citral
X_2_: Terpene amount (mg)	10	30
Responses	Constraints	
Y_1_: EE (%)	Maximize	
Y_2_: PS (nm)	Minimize	
Y_3_: PDI	Minimize	

All formulations contain 10 mg FTN.

### 2.3 Characterization of fenticonazole-terpesomes

#### 2.3.1 Entrapment efficiency% and drug loading

The TP dispersions were centrifuged at 20,000 rpm for 1 h at 4 °C using a cooling centrifuge (Sigma 3K30, Germany). The obtained pellet was then lysed, and the drug content was quantified using a UV–visible spectrophotometer (Shimadzu UV-1650, Japan) at 252 nm. The entrapment efficiency (EE%) was calculated using the direct determination method (Abdellatif et al., 2017). The drug loading of different formulations was calculated as reported previously (Abdellatif et al., 2025).

EE% is calculated using Equation 1 as follows:

$$\text{EE}\%=\left(\frac{\text{Entrapped}\;\text{FTN}}{\text{Total}\;\text{FTN}\;\text{concentration}}\right)\;x\;100.$$ (1)

Drug loading % is calculated using Equation 2 as follows:

$$\text{EE}\%=\left(\frac{\text{Entrapped}\;\text{amount}\;\text{of}\;\text{FTN}}{\text{Total}\;\text{amount}\;\text{of}\;\text{lipid}}\right)\;x\;100.$$ (2)

#### 2.3.2 Particle size, polydispersity index, and zeta potential

The particle size (PS), polydispersity index (PDI), and zeta potential (ZP) of TPs of the prepared TPs were measured using a Zetasizer (Malvern Ltd., United Kingdom) (Yousry et al., 2023).

#### 2.3.3 Experimental design construction

A 3^1^.2^1^ factorial design was created in Design Expert to estimate the impact of the factors on TP formulation. Two variables were examined in this study: the type of terpene (X_1_) and its amount (X_2_), which served as the independent factors. The corresponding dependent responses were the entrapment efficiency (EE%, Y_1_), particle size (PS, Y_2_), and polydispersity index (PDI, Y_3_), as shown in Table 1. The experimental design aimed to identify the formulation that achieved the highest EE% while also minimizing the PS and PDI values. The formulation demonstrating the highest overall desirability was chosen for subsequent characterization.

#### 2.3.4 Optimization of the selected fenticonazole-terpesomes

Preliminary experiments were carried out to determine the optimal parameters using different concentrations of chitosan as the coating material. Chitosan was used in the construction of the selected TPs from the previously designed formulations during the hydration step. To enhance the mucoadhesive properties of TPs, chitosan-coated TPs were developed. In summary, 10 mL of water containing 1% glacial acetic acid was used to dissolve chitosan (0.1, 0.3, and 0.6% w/v) (Supplementary Table 1), which was then added to the same volume of the previously prepared TPs and mixed for 15 min. In addition, Gelucire (44/14) (10 mg) was added to the organic solvent (methanol) in the round flask as a PEG-containing surfactant to obtain the optimum CCPTs.

#### 2.3.5 Transmission electron microscope

A transmission electron microscope (TEM; JEOL, Japan) was used to investigate the morphology of the optimum CCPTs. A thin layer of the vesicular dispersion was applied to a grid, which was then stained and photographed (Yousry et al., 2020; Mohamed et al., 2024).

#### 2.3.6 *In vitro* release

Dissolution apparatus II equipment (Hainburg, Germany) was utilized to assess the *in vitro* release over 6 h at 37 °C (Geng et al., 2011). One mg of FTN from the optimum CCPTs was precisely placed in a cylindrical plastic chamber with a 0.76 cm^2^ permeation area. A cellulose membrane with a molecular weight cutoff of 12,000–14,000 was used to seal one end of the diffusion chamber, while the opposite end was connected to the shaft of the dissolution apparatus. The receptor compartment was filled with 50 mL of phosphate buffer (pH 7.4). At predetermined intervals of 1, 2, 3, 4, 5, and 6 h, samples were withdrawn and analyzed using a UV–visible spectrophotometer at λ_max_ of 252 nm.

#### 2.3.7 Confocal laser scanning microscopy study

To track the movement of the optimum CCPTs across the different layers of the urinary bladder, the formulation was prepared as described, except that FTN was replaced with FDA at 1% (w/v) (Fang, et al., 2010). Urinary bladder tissue was placed in diffusion chambers with the same specifications as those used in the *in vitro* release. FDA-CCPTs were positioned on the surface of the urinary bladder and left in place for 6 hours to simulate the application of the optimum CCPTs in contact with the bladder. The bladder tissues were then fixed, sectioned using a microtome, and examined for fluorescence (Wetzlar, Germany). The slides were then examined using an inverted microscope (Carl Zeiss, Germany) (Eltabeeb et al., 2025).

#### 2.3.8 Assessment of the mucoadhesion property of the optimum terpesomes

The mucoadhesion property of the selected formulation (TPs) and the optimum formula after coating (CCPTs) were examined by mixing each with porcine mucin in equal amounts. The mixture was then stirred for an additional 5 min and kept overnight. A Zetasizer was used to measure the zeta potential of both mucin alone and the vesicles (Abdellatif et al., 2020).

#### 2.3.9 Stability study

The stability of the optimum formula was evaluated to determine the ability of the CCPTs to undergo aggregation or any other variations. The optimum CCPTs were stored for 3 months at room temperature. After the specified time, the stored formulation was analyzed, and the results were compared with the freshly prepared formulation (Ahmed et al., 2025a).

#### 2.3.10 FT-IR spectroscopy

FT-IR analysis was performed for the individual components, including FTN, PC, Gelucire (44/14), cineole, chitosan, their physical mixture, the optimum formula, and the corresponding drug-free (blank) formulation using an FT-IR spectrophotometer (Bruker, United Kingdom). This analysis aimed to detect any possible interactions between FTN and the formulation excipients. Spectra were recorded at 25 °C over 4000–500 cm^-1^ after carefully drying the samples and compressing them into potassium bromide (KBr) discs (Ahmed et al., 2025b).

#### 2.3.11 *In vitro* lanosterol 14α-demethylase inhibitory assay

Using fluconazole as a reference, the inhibitory activity of fenticonazole nitrate, chitosan, cineole, and Gelucire (44/14) against lanosterol 14α-demethylase (CYP51) was evaluated. Cytochrome P450 (CYP) isoform CYP1A1 is competitively suppressed by the fluorescent substrate 7-ethoxyresorufin (7-ER) (IC_50_ = 0.1 μM). CYP1A1 activity was measured using the fluorescence of resorufin, which was liberated by enzymatic cleavage by CYP1A1. Resorufin has consecutive excitation and emission peaks of λ_max_ 572/604 nm. Since resorufin and 7-ER are sensitive to light, the protocol stages were carried out under yellow light to preserve the integrity of the stock solutions. 7-ER and CYP51 bactosomes were incubated in a phosphate buffer containing magnesium chloride in a black 96-well plate. A measure of 40 μL of a 5x NADPH-producing system was added to initiate the reactions. Using detection wavelengths chosen to reduce interference from NADPH and 7 ER, resorufin production was measured fluorometrically (Hassan et al., 2022).

### 2.4 Computational study

The structure of 14α-demethylase CYP51 (PDB ID: 5V5Z) was acquired from the RCSB PDB database. The protein structure was prepared by removing water molecules and the co-crystallized ligand 1 YN (HEM) before docking the integrated congeners into its active site.

In brief, molecules were 3D-constructed utilizing the builder module and isomeric SMILES strings obtained from the PubChem database for the investigated compounds and metabolites (PubChem CID: 51755, 65167, 2758, 71853, and 135305178 for FTN, PC, cineole, chitosan, and Gelucire (44/14), respectively) (Albash et al., 2021a). Gelucire 44/14 is a mixture of esterified molecules resembling the analogs of lauroyl PEG-32 glycerides; therefore, structurally diverse molecules were selected as representative components of this nanoformulation additive. Each molecule was minimized separately down to a low energy gradient (RMS 0.00001 kcal/mol.A^2^) using the Amber10:EHT force field, which has been optimally parametrized for small molecules. Docking flow was performed using PC as the target molecule and both the drug (FTN) and formulation components as the ligands. The triangular matcher approach was conducted to generate the final target–ligand complex, where different conformations were initially ranked via the London/dG scores, and subsequently, the top 10 scored poses were refined through an energy minimization stage inside the target interface. Rescoring of the obtained binding modes was carried out using the Generalized Born-solvation_VI/weighted-surface area/dG relying on van der Waals score, Coulomb potentials, current partial charges, solvation electrostatic potentials, and exposure-weighted surface area (Vilar et al., 2008). Optimum hydrogen bond interaction was set at cut-offs of 3.0 Å bond length and 20° angle (De Souza et al., 2019; Albuquerque, et al., 2020), whereas the interactions were assigned at ≤ 5 Å using the MOE ligand interaction module and Schrödinger-PyMOL (New York, United States) built-in measurement.

The top-docked target–ligand complex then proceeded through an all-atom molecular dynamics (MD) simulation to investigate the complex’s thermodynamic stability under the influence of solvent, temperature, and pressure. Initially, the complex was minimized using the explicit Amber10:EHT force field to a very low gradient (RMS 0.0005 kcal/mol.A^2^) and then solvated within a 3D-cubic box (45 × 45 × 45 Å) containing 3329 molecules of water. Periodic boundary conditions were applied in all dimensions, maintaining a 10 Å non-bounded cut-off. The constructed system (Table 2) was then minimized (500 ps) and subsequently equilibrated (500 ps for each step) under an initial constant number, volume, and 310 K temperature (NVT) ensemble using the Nosé–Hoover–Andersen equations of motion. An additional equilibration stage (500 ps) was conducted at a constant number, pressure (101 kPa), and 310 K temperature (NPT) ensembles to avoid the generation of any artifacts when starting the MD simulation immediately after the NVT ensemble. Finally, the minimized/equilibrated system was allowed to run for 1 ns under NPT, where all constraints were removed. Analysis of the MD was carried out using the MOE calculator for estimating the interaction between FTN and PC.

**TABLE 2:** Atomic composition of the FTN–PC formulation.

Solvation state	Atomic composition (No. of atoms)						
	FTN	PC	Cineole	Chitosan	Gelucire 44/14 multi-component	Water	Total
100% water	50	134	29	207	199	3 x 3329	10,446

### 2.5 *In vitro* antifungal assessment

The broth microdilution technique was used to quantify the minimum inhibitory concentration (MIC). Each well of a sterile 96-well plate was filled with 100 μL of two-fold strength Sabouraud dextrose broth. The first well in each row was then filled with 100 μL of FTN suspension. The FTN suspension was serially diluted two times from row to row until the ninth row. After that, 10 μL of the *Candida albicans ATCC 60193* (10^7^ CFU/mL) was added to the wells. One row was utilized as a control for growth, while another row served as a control for sterility. The plates were incubated in an aerobic atmosphere for 24 h.

### 2.6 *In vivo* urinary bladder infection model

The *in vivo* studies were performed in accordance with the guidelines of the Faculty of Pharmacy, The British University in Egypt, under approval number Ex-2301. Male Wistar rats, aged 6–7 weeks (155 ± 16 g), were used to establish a *C. albicans* urinary bladder infection model as previously described, with modifications (Andes, et al., 2004; Eltabeeb, et al., 2024). Before infection, animals received hormonal and immunomodulatory pretreatments to facilitate fungal colonization. This preconditioning helped maintain an optimal physiological environment that favored pathogen persistence and mimicked the host conditions commonly associated with clinical bladder infections. Fungal colonization was established by intravesical inoculation of rats using a catheter with a suspension of *C. albicans* ATCC 10231 (7–15 × 10^8^ CFU) once daily for four consecutive days to induce bladder infection. After confirmation of infection, the animals were randomly allocated into three groups (n = 6). Treatments were administered intravesically (250 µL of the optimum formulation and the free drug suspension) 24 h after the final inoculation using a sterile catheter. The first group served as a positive control, the second received the free drug, and the third was treated with the optimum formulation. After 72 h, samples were collected aseptically and serially diluted in phosphate buffer before plating on Sabouraud dextrose agar to determine fungal counts. The results obtained from different groups were statistically compared to evaluate the antifungal pharmacological effect of the tested formulations.

### 2.7 Histopathological examination

Three male Wistar rats from each group (normal group, optimum formula (CCPTs), and FTN suspension) were euthanized and gently put down after urinary bladder administration by catheter to gather tissue samples for histology and assess the local tissue responses and signs of inflammation. One-centimeter-sized tissue samples from the urinary bladder were first preserved in 10% formalin, dehydrated in ethanol, and then embedded in paraffin. Hematoxylin and eosin-stained tissue sections, measuring 3–5 μm in thickness, were examined under a Leica DM 5000B light microscope in Wetzlar, Germany (Eltabeeb et al., 2024).

## 3 Results and discussion

### 3.1 3^1^.2^1^ factorial design

#### 3.1.1 Entrapment efficiency (%)

The quantity of drug encapsulated within the nanoparticles relative to the total quantity of drug added is expressed as the EE% (Shah et al., 2014). The EE% of FTN-loaded TPs ranged from 69.76% ± 4.18% to 85.56% ± 1.63% (Table 3), while the drug loading percentage ranged from 7.02% ± 1.22% to 8.55% ± 1.34%.

**TABLE 3:** 3^1^.2^1^ full factorial design for FTN-TPs.

	Terpene type	Terpene amount (mg)	EE%	PS (nm)	PDI	ZP (mV)	Drug loading (%)
F1	Cineole	10	72.77 ± 5.08	517.60 ± 12.92	0.56 ± 0.01	8.03 ± 0.62	7.94 ± 1.23
F2	Cineole	30	69.76 ± 4.18	212.30 ± 1.83	0.42 ± 0.002	14.10 ± 3.93	7.28 ± 1.89
F3	Menthol	10	77.95 ± 6.33	550.98 ± 113.28	0.54 ± 0.003	11.90 ± 0.79	7.29 ± 0.56
F4	Menthol	30	72.21 ± 5.16	390.98 ± 10.12	0.46 ± 0.004	15.10 ± 0.24	7.02 ± 1.22
F5	Citral	10	85.56 ± 1.63	630.20 ± 42.7	0.72 ± 0.01	13.7 ± 1.16	8.55 ± 1.34
F6	Citral	30	77.30 ± 1.35	523.4 ± 27.56	0.78 ± 0.12	3.43 ± 0.40	7.73 ± 1.24

**FIGURE 1:**
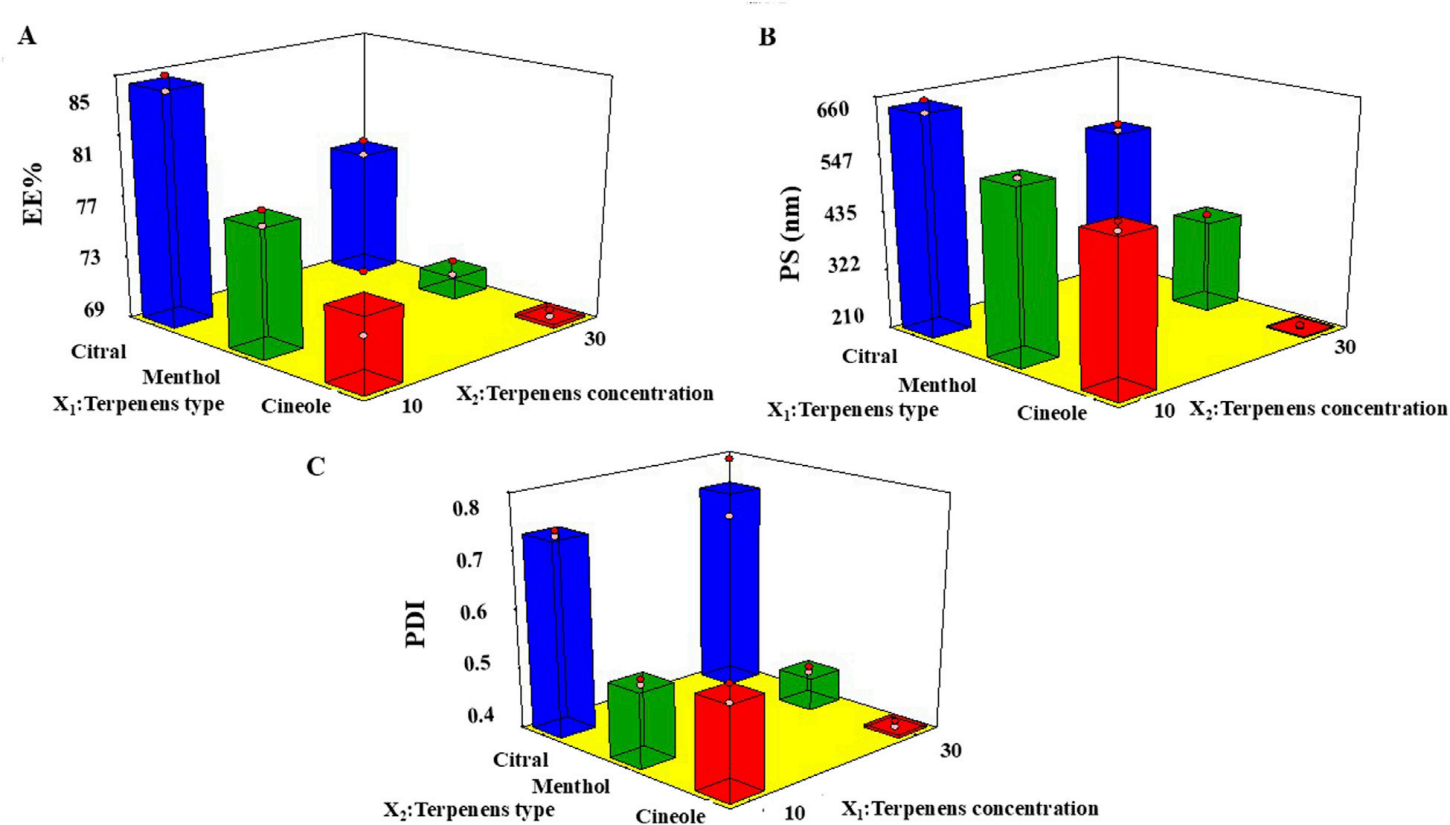
Response 3D plots for the effect of terpene amount (X_1_) and terpene type (X_2_) on **(A)** EE%, **(B)** PS, and **(C)** PDI of FTN-TPs.

The relatively high EE% of FTN may be ascribed to the lipophilic characteristics of the TP matrix, which facilitates the incorporation of lipophilic drugs such as FTN. In this study, the terpene type (X_1_) showed a significant effect (*p* < 0.0001) on the EE% of FTN (Figure 1A). The highest EE% values were observed in formulations containing citral, followed by menthol, and then cineole. This trend corresponds with the degree of lipophilicity of the terpenes, as reflected by their log P values of 3.45, 3.2, and 2.97 for citral, menthol, and cineole, respectively (Albash et al., 2021b).

Similarly, a previous study reported that higher EE% values for lipophilic drugs such as dapsone were achieved when terpenes with greater lipophilicity were used (El-Nabarawi et al., 2018). Regarding the terpene amount (X_2_), an inverse relationship was observed, where the EE% significantly decreased (*p* < 0.0001) with increasing terpene amount (Figure 1A). This reduction may be explained by the formation of pores in the lipid bilayer at higher terpene levels, which could compromise vesicle integrity and lower EE%. Additionally, excessive terpene content might increase PC fluidity around the C_16_ atom of the acyl chains, thereby affecting the vesicles’ ability to entrap FTN (Teaima et al., 2022).

The influence of the investigated factors on EE% can be described using the following coded equation:

$$\text{EE}\%=75.54-3.13X_{2}-3.92X_{1\;}\left[1\right]-1.54X_{1\;}\left[2\right]+0.75X_{2}X_{1}\left[1\right]-0.37X_{2}X_{1}\left[2\right],$$

where X_1_ represents the terpene type and X_2_ represents the terpene amount.

#### 3.1.2 Particle size

According to previous studies, the PS of the TPs is a critical parameter for determining their suitability for intravesical application. Intravesical drug delivery often fails to achieve sufficient drug concentrations due to the urothelium, which consists of six to seven cellular layers, acting as a strong barrier against drug permeation. Therefore, nanosized carriers with smaller PS hold great promise for improving intravesical drug delivery as they enhance the drug penetration through the urothelium. In addition, smaller particles possess a higher surface-to-volume ratio, which can further improve the transvesical absorption of encapsulated drugs (Şenyiğit et al., 2015).

As shown in Table 3, the PS of the formulated TPs ranged from 212.00 ± 1.00 nm to 630.00 ± 42.00 nm. ANOVA results revealed that both the type of terpene (X_1_) and its amount (X_2_) significantly influenced the PS. As illustrated in Figure 1B, X_1_ had a highly significant effect (*p* < 0.0001), where TPs prepared using citral exhibited the largest PS compared to those containing menthol or cineole. This finding aligns with the EE% results as citral-based formulations also showed the highest EE%. Previous reports have suggested that higher EE% values may correspond to larger PS, likely because the vesicular bilayer expands as more drug molecules occupy the hydrophobic regions of the vesicle. Hence, TPs with higher EE% tend to have larger PS values.

Moreover, the terpene amount (X_2_) significantly reduced the PS (*p* < 0.0001). This could be attributed to the higher terpene content decreasing the viscosity and interfacial tension of the internal phase of the dispersion medium, thereby reducing the likelihood of Ostwald ripening and leading to smaller, more stable vesicles (Asadinezhad et al., 2019).

The influence of the investigated factors on PS can be described using the following coded equation:

$$\text{PS }\left(\text{nm}\right)=472.50-94.83X_{2}-111.50X_{1}\left[1\right]-2.00X_{1}\left[2\right]-53.67X_{2}X_{1}\left[1\right]+14.83X_{2}X_{1}\left[2\right],$$

where X_1_ represents the terpene type and X_2_ represents the terpene amount.

#### 3.1.3 Polydispersity index

As presented in Table 3, the PDI of all formulations ranged from 0.420 ± 0.002 to 0.780 ± 0.12. These results indicate that TPs were polydisperse yet remained within acceptable limits (Teaima et al., 2022). Both the terpene type (X_1_) and terpene amount (X_2_) significantly influenced the PDI, with *p*-values of 0.0084 and <0.0001, respectively (Figure 1C).

A direct relationship was observed between PS and PDI, where vesicles exhibiting larger PS also showed higher PDI values. This relationship is consistent with previous findings reported by Tawfik et al. (2020), suggesting that larger vesicles are more likely to display greater size variability within the dispersion.

The influence of the investigated factors on PDI can be studied using the following coded equation:

$$\text{PDI}=0.58-0.034X_{2}-0.087X_{1}\left[1\right]-0.078X_{1}\left[2\right]-0.041X_{2}X_{1}\left[1\right]-0.000833X_{2}X_{1}\left[2\right],$$

where X_1_ represents the terpene type and X_2_ represents the terpene amount.

#### 3.1.4 Zeta potential

ZP values of all the prepared TPs exhibited positive charges, ranging from 3.43 ± 0.40 to 15.10 ± 0.24 mV, as shown in Table 3. The low positive ZP values may be attributed to the presence of nitrogen atoms in the FTN, which impart a positive charge to the otherwise neutral PC used (Younes et al., 2018). ANOVA results showed that both X_1_ and X_2_ had no significant influence on the ZP of the TPs with a *p*-value of ˃ 0.05; hence, ZP was removed from the criteria for selection.

### 3.2 Determination of the selected terpesomes

The selected TP formulation was determined through numerical evaluation of the experimental design. The optimization process targeted the formulation that achieved the highest EE% along with the lowest PS and PDI (Table 4). Based on the desirability function outcomes, formulation F2, containing 30 mg of cineole, was identified as the selected and subsequently subjected to comprehensive characterization.

**TABLE 4:** Output data of the full factorial design (3^1^.2^1^) analysis of TPs.

Responses	EE%	PS (nm)	PDI
Adequate precision	16.60	64.26	15.43
Adjusted *R* ^2^	0.98	0.99	0.97
Predicted *R* ^*2*^	0.94	0.99	0.94
Significant factors	X_1_, X_2_	X_1_, X_2_	X_1_, X_2_
Predicted value of F2	69.25	212.50	0.41
Observed value of F2	69.67	212.30	0.42

### 3.3 Optimization of the selected terpesomes

Bioadhesive polymers are employed to deliver drugs to a particular area of the body over a prolonged period, and these polymers adhere to the mucin or epithelial surface to facilitate drug delivery. Several studies in the urological field have reported the use of a wide range of bioadhesive polymers to extend the retention time of drugs in the bladder (Şenyiğit et al., 2015). Among these bioadhesive polymers, chitosan, a natural copolymer, exhibits several advantageous characteristics, including strong bioadhesion, permeation-enhancing properties, and controlled drug release, attributed to its cationic nature arising from primary amino groups (Bernkop-Schnürch and Dünnhaupt, 2012).

Accordingly, the selected TP formulation (F2, containing 30 mg of cineole) was further optimized by incorporating chitosan (0.3%) as a cationic coating agent. The introduction of a positive surface charge aimed to enhance the formulation’s suitability for urinary bladder delivery, since positively charged vesicles can promote electrostatic interactions with the negative mucin layer, thereby improving adhesion and deposition on the bladder surface. In addition, Gelucire 44/14 (10 mg) was added as a PEGylated surfactant, since previous reports indicated that PEGylation of the formulated vesicles can alter their interfacial characteristics, influencing deposition and residence time at the application site (Abdelbari et al., 2023).

As shown in Table 3, F2 was selected based on the highest possible ZP value and indicated a low positive charge. The ZP of F2 was found to be 14.10 ± 3.93 mV (low positive ZP).

In the current study, the PEGylated optimized F2-coated with chitosan showed an increase in the EE% and ZP values without significant changes in PS (227.06 ± 4.73 nm) or PDI (0.63 ± 0.07). EE% was increased to 84.49% ± 2.47%, possibly due to the high viscosity that occurred upon the addition of chitosan, lesser diffusion of the drug from vesicles, and thus a higher degree of encapsulation (Ameeduzzafar et al., 2020). Furthermore, ZP increased to 32.65 ± 2.29 mV, which is expected to augment the vesicle’s stability as the chance for vesicles’ agglomeration will be slight for charged particles having a ZP ≥ 30 mV due to electrostatic repulsion (Arneth et al., 2025). Furthermore, the presence of a PEGylated surfactant may have modified the vesicles’ interfacial characteristics, boosting the deformability of the vesicles. This enhanced vesicular deformability is believed to promote deeper penetration through the mucus layer by the vesicles (Qadri et al., 2017). Moreover, the vesicles may penetrate the mucus layer more effectively, enhancing drug distribution and retention at the target site while minimizing rapid clearance from superficial mucus layers (Abdellatif et al., 2020).

#### 3.3.1 Transmission electron microscope

**FIGURE 2:**
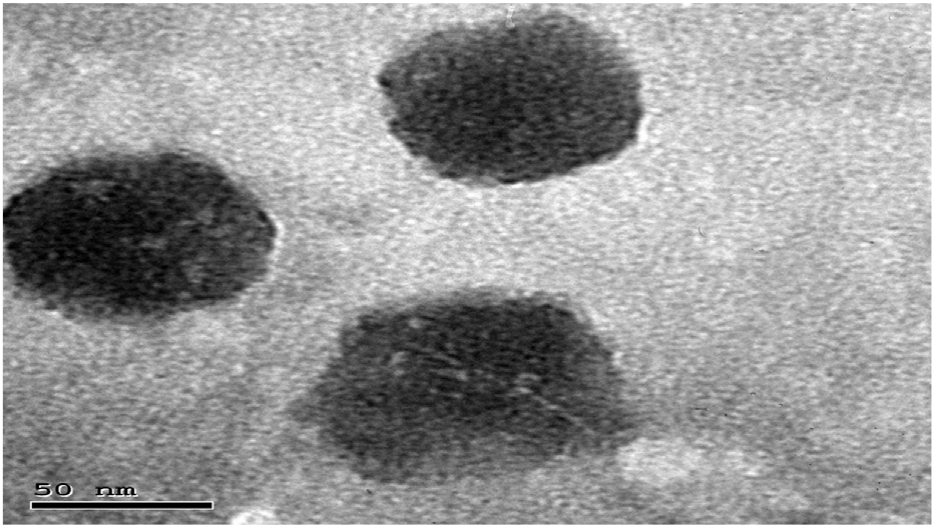
Morphology of the optimum CCPT.

Figure 2 shows the morphological characteristics of the optimized formula. The TEM image revealed that the optimized formula exhibited spherical vesicles with uniform distribution. These findings are in agreement with previous reports (Fahmy, et al., 2025).

#### 3.3.2 *In vitro* release

**FIGURE 3:**
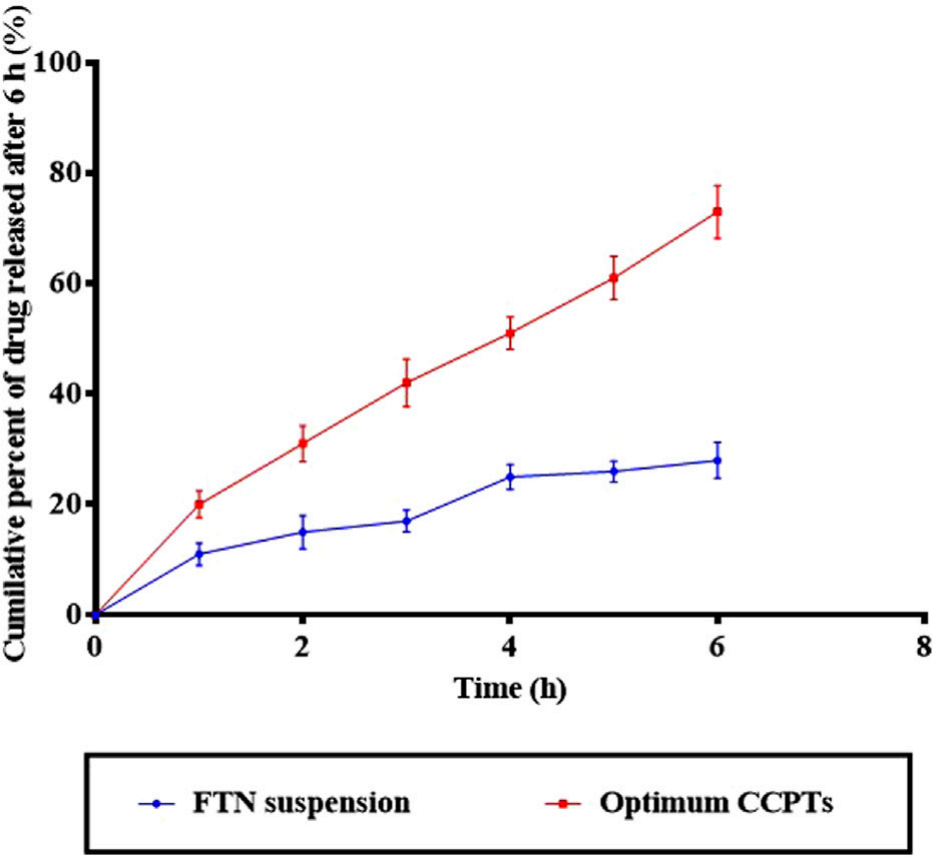
Release study of the optimum CCPTs compared to FTN suspension.

The release profile of FTN from the optimized FTN-CCPTs was related to that of the pure FTN suspension, as illustrated in Figure 3. The pure FTN suspension exhibited a low cumulative drug release of 28.00% ± 2.90% after 6 h, whereas the optimized FTN-CCPTs showed a significantly higher release of 73.00% ± 3.70% over the same period. The enhanced FTN release from the optimized formulation may be attributed to the solubilizing capacity of Gelucire 44/14, which possesses surface-active properties that improve the solubility of sparingly water-soluble drugs. This observation is consistent with the results of Mura et al. (2021), who reported that solid lipid nanoparticles prepared using Gelucire 44/14 exhibited enhanced drug release due to their amphiphilic nature.

#### 3.3.3 Confocal laser scanning microscopy

**FIGURE 4:**
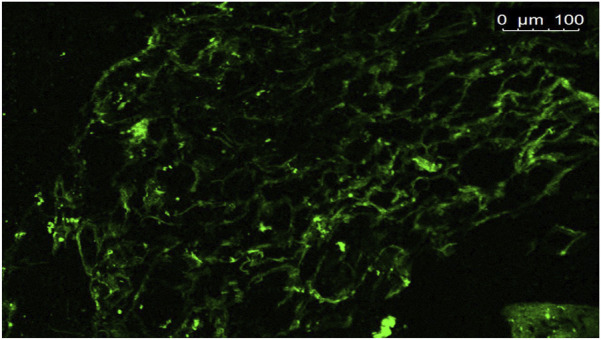
Tile scan confocal laser microscope photomicrograph of longitudinal section in rat urinary bladder with FDA loaded CCPTs.

Images acquired via confocal laser scanning microscopy (CLSM) (Figure 4) showed that the FDA-loaded optimized formula presented fluorescence accumulation across various bladder layers. Scans were taken from sections that provided data on the deposition of fluoro-labeled CCPTs in bladder tissues, confirming the interaction and distribution of the formulation observed by CLSM. The fluoro-labeled optimum formula showed homogeneous diffusion in the bladder tissues. This result can be credited to the small PS of the fluoro-labeled optimized formula, which may facilitate the distribution of CCPTs within the bladder tissue. In addition, the strong bioadhesive properties and prolonged retention time achieved through chitosan incorporation, as discussed earlier, further support this outcome. Moreover, PEGylation enhanced adhesion to the bladder through the development of covalent bonds with the thiol present in the mucin of the bladder epithelium (Kaldybekov et al., 2018).

#### 3.3.4 Assessment of the mucoadhesion property

After mixing mucin with the selected formulation (F2) and the optimized formulation (CCPTs), the ZP changed by −9.90 ± 0.13 mV. The complexes formed between the formulations and mucin exhibited positive ZP values, indicating that the positive charges of the formulations adhered to the mucin surface, thereby neutralizing its inherent negative charge. Previous studies have reported that the adhesion of a mucoadhesive substance can alter the surface characteristics of mucin (Eltabeeb et al., 2025). The mucoadhesive selected formulation (F2) caused a shift in mucin ZP values from negative to positive, reaching 3.26 ± 1.56 mV, whereas the optimized CCPTs demonstrated a more pronounced shift to 26.23 ± 2.25 mV. This substantial increase in ZP values for the CCPTs indicates a stronger mucoadhesive capability compared to the selected F2 formulation (Abdellatif et al., 2020).

#### 3.3.5 Stability study

Visual inspection of the optimum CCPT formulation revealed no signs of sedimentation during the storage period. The recorded values for EE%, PS, PDI, and ZP were 84.49% ± 2.47%, 291.45 ± 34.32 nm, 0.49 ± 0.01, and 30.5 ± 3.89 mV, respectively, with no significant deviation from the freshly prepared formulation (*p* > 0.05). These results indicate the physical stability of the optimum formulation, which could be attributed to the presence of chitosan. The positively charged chitosan coating likely enhanced electrostatic repulsion between vesicles, thereby preventing agglomeration and fusion and contributing to the overall stability of the CCPTs (Salem, et al., 2019).

#### 3.3.6 FT-IR spectroscopy

**FIGURE 5:**
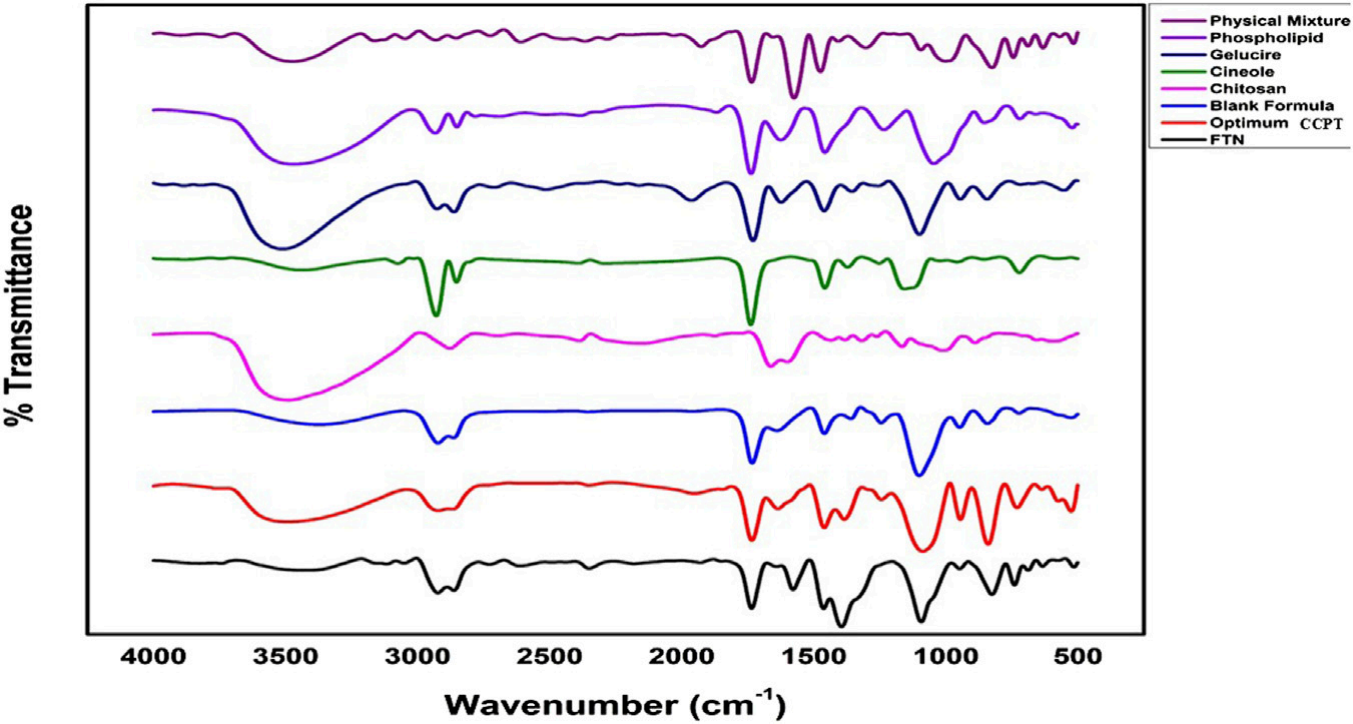
FT-IR spectra of FTN, excipient, physical mixture, and the optimum formula.

The FT-IR spectrum of FTN (Figure 5) exhibited characteristic absorption bands corresponding to C–N stretching at 1581.63 cm^-1^, aromatic C–C stretching at 1469.76 cm^-1^, C–O–C ether stretching at 1091.71 cm^-1^, and C–Cl stretching at 794.67 cm^-1^ (Nemr, et al., 2024). Furthermore, comparison of the FT-IR spectra of FTN with that of its physical mixture revealed the presence of all the characteristic absorption bands of the drug within the mixture, suggesting the absence of any significant chemical interactions between FTN and the formulation excipients. In contrast, the characteristic drug bands disappeared from the IR spectrum of the optimum CCPTs, suggesting efficient encapsulation of the drug within the nanovesicles (Khan et al., 2019; Ahmed et al., 2022b). Moreover, the IR spectra of the optimum CCPTs and the blank formulation (without drug) were nearly identical, further confirming the successful encapsulation.

#### 3.3.7 *In vitro* lanosterol 14α-demethylase inhibitory assay

Fenticonazole nitrate, chitosan, cineole, and Gelucire (44/14) against lanosterol 14α-demethylase (CYP51) were evaluated employing fluconazole as a reference using the CYP51 inhibitory kit assay. Fenticonazole nitrate, chitosan, cineole, and Gelucire (44/14) effectively inhibited *Candida* lanosterol 14α-demethylase with IC_50_ values of 1.04 ± 0.13, 3.36 ± 0.02, 4.67 ± 0.03, and 4.98 ± 0.01 µM, respectively, compared to the fluconazole IC_50_ value of 0.753 ± 0.03 µM. These findings suggest that fenticonazole nitrate is a potent CYP51 inhibitor comparable to fluconazole, while chitosan, cineole, and Gelucire (44/14) exhibit secondary or complementary inhibitory effects that may enhance overall antifungal efficacy when formulated together.

### 3.4 Computational study

Docking analysis of fenticonazole, which presented a binding affinity of −44.59 kcal/mol. A key interaction is observed between the ligand’s aromatic ring and Cys470, forming a π–sulfur interaction, which plays an important role in anchoring the ligand close to the catalytic region. Additionally, the ligand establishes a π–π stacking interaction with Phe475, enhancing the stability of the aromatic core of the compound inside the binding site. Hydrophobic interactions also play a major role in maintaining the ligand fit. Residues such as Ala476, Ile471, and Tyr132 form π–alkyl and sulfur–alkyl contacts with the ligand’s hydrophobic fragments. Furthermore, Lys143 interacts with the ligand through π–cation interactions (Supplementary Figure 1).

**FIGURE 6:**
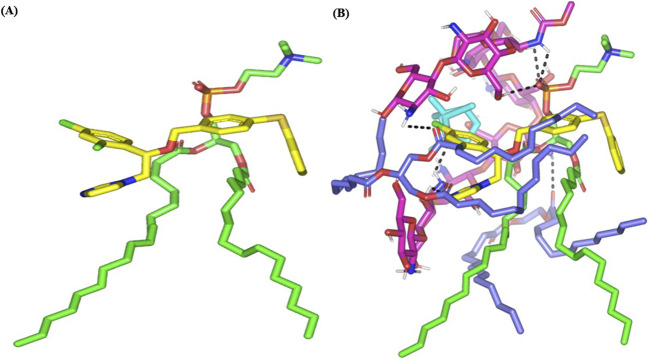
Predicted binding modes of the FTN–PC docked complex. Three-dimensional representation of the FTN (yellow) being loaded on the PC interface (green), in the absence **(A)** and in combination **(B)** with the formulation additives: chitosan (magenta), cineole (cyan), and Gelucire 44/14 multiple components (blue). Chitosan served as a supportive platform for favored anchoring of formulation additives on the drug–PC complex. The polar interactions, discussed within the text, are depicted as black dashed lines.

Molecular docking revealed the FTN–PC interaction, where the drug exhibited a favored orientation near the phosphate head of the PC while adopting an extended conformation toward the hydrophobic acyl chains of the PC (Figure 6A) (Abd-Elsalam et al., 2018; Farag et al., 2021). Depiction of the extended FTN conformation around the PC acyl chains can be attributed to the dominant van der Waals energy contributions between the FTN and PC complex, mediated by the significant hydrophobic nature of the investigated drug (logP 6.7; four aromatic rings with one ring being double chlorinated). Additionally, the absence of complementary hydrogen bond donors capable of satisfying the electrostatic potential generated by the hydrogen bond acceptors of either the drug or PC resulted in a predominant nonpolar energy contribution. Despite the significant hydrophobic-driven binding between both molecules, this was not enough to build a stable FTN–PC complex with a favorable stability profile. Consequently, a relatively low docking score (−4.52 kcal/mol) was assigned to the FTN anchoring at the PC molecule.

Molecular investigation of the FTN–PC, combined with formulation components cineole, chitosan, and Gelucire 44/14, revealed enhanced stabilization and more favorable binding interactions (Figure 6B). The molecular conformation and high polarity of chitosan enabled the formation of a flexible network around the FTN–PC complex, promoting multiple strong polar interactions that contributed to the stability of the system. Two consecutive 1→4 β-linked glucosamine residues (M_2_ and M_3_) established hydrogen bonds with the phosphate group of PC [–OPO(O)OH], established via the C_3_–OH, C_6_–OH, and C_2_–NH_2_ groups of chitosan, with respective bond distances and angles of 2.3 Å/131.8°, 2.3 Å/166.3°, and 2.5 Å/121.6°. Additionally, the terminal glucosamine residue (M_8_) of chitosan formed a hydrogen bond with the pyrazole ring of FTN via its C_3_–OH group (2.7 Å/134.2°). Notably, chitosan acted as a structural bridge, facilitating the favorable anchoring of cineole and Gelucire 44/14 molecules onto the FTN–PC complex. Cineole binding was stabilized through hydrogen bonding between the C_2_–NH_2_ of chitosan (M8) and the bridged oxygen atom of cineole (2.5 Å/139.9°), allowing the hydrophobic cyclohexane moiety of cineole to align closely (∼4.0 Å) with the chlorinated aromatic ring of FTN, thereby promoting strong hydrophobic interactions.

Stability of Gelucire 44/14 multiple molecules at the drug–PC complex was facilitated by polar interaction between its ester carbonyl groups and the hydroxy functionality at the chitosan sugar monomers (<2.0 Å/> 155.2°). Furthermore, Gelucire 44/14 molecules exhibited close-range hydrophobic contacts with the extended non-polar acyl chain of the docked PC molecules. Notably, the long hydrophobic chain of the lauroyl derivatives was predicted to adopt a favored orientation along the carbon skeleton of the FTN molecule, enabling relevant van der Waals contacts (<4.0 Å). In this context, incorporation of the three components resulted in a stable FTN–PC complex, characterized by a network of electrostatic and hydrophobic interactions. This structural stabilization was reflected in the favorable docking energy value of −12.03 kcal/mol. The docking results further elucidated the enhanced formulation features detected upon inclusion of chitosan, which acted as a carrier matrix facilitating FTN association with the PC molecule, thereby enhancing drug solubilization and overall system stability.

**FIGURE 7:**
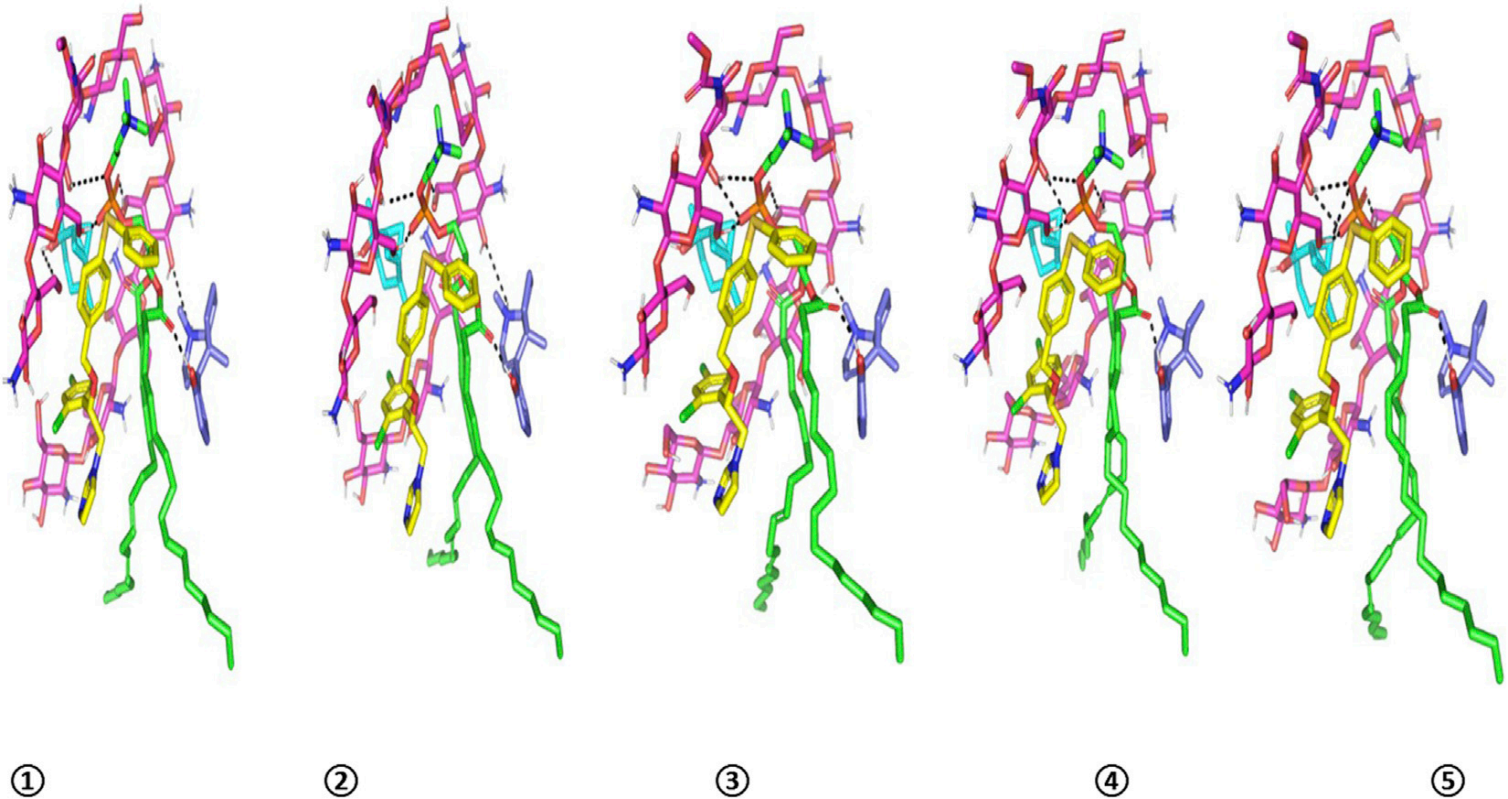
Conformation alteration-time evolution of FTN–PC formulation additive heterocomplex throughout the all-atom MD simulation within 100% aqueous system. The thermodynamic movements: FTN (yellow), PC (green), chitosan (magenta), cineole (cyan), and Gelucire 44/14 (blue) at different snapshots ① 0.2 ns, ② 0.4 ns; ③ 0.6 ns; ④ 0.8 ns; and ⑤ 1 ns.

Investigating the thermodynamic stability and dispersion behavior of the docked drug–PC complex within the formulation’s final solvent (100% water) was performed through an explicit MD simulation study. Notably, it demonstrated great stability for the FTN–PC complex across the whole MD simulation run (Figure 7). An average sum-over-states binding energy (−148.85 ± 3.83 kcal/mol) was obtained for the drug in complex with the formulated PC carrier. The van der Waals potentials dominated the binding interaction energy contribution, while several polar contacts were maintained along the whole MD simulation. Predominant hydrophobic van der Waals interactions highlighted the advent role of the PC lipophilic acyl scaffold and those of the Gelucire 44/14 multiple molecules for the FTN nanoformulation. It is worth mentioning that a minimal structural shift was observed for each simulated molecule until the end of the MD simulation run, showing a root-mean-squared deviation (RMSD) below 2.0 Å. This dynamic behavior illustrated limited drug and nanoformulation additive orientation drift owing to the combined favorable non-polar binding interactions. The extended hydrogen bond network between the hydrophilic chitosan’s OH functionalities and other nanoformulation components were maintained across the entire simulation run. These latter hydrogen bond pairs illustrated optimal hydrogen bond distances and angles (<2.5 Å/> 135.0°) the thing which maintained tight closeness between the formulation carrier additives and the drug.

**FIGURE 8:**
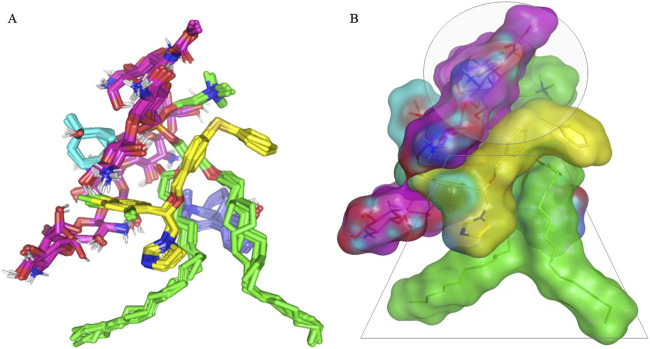
Overlay of FTN–PC formulation additive complex across MD simulation frames (left panel) and **(A)** molecular surface 3D representation of the inverted cone micellar configuration within 100% aqueous solvation system (right panel). **(B)** Molecular surface and stick 3D representations were illustrated in colors being previously assigned for the optimum formulation; yellow, green, magenta, cyan, and blue are for FTN, PC, chitosan, cineole, and Gelucire 44/14, respectively.

Regarding the spatial conformation of the PC lipophilic chains, interesting findings were depicted. The conserved polar contacts at the phosphate group of PC carriers and the maintained strong hydrogen bond pairing via the PC acyl group and multiple Gelucire 44/14 multiple permitted the two PC hydrophobic acyl tails to be pulled away from each other. This dynamic behavior produced an open-compass conformation for the PC tails, which would have increased the volume of the hydrophobic chain. In contrast, a small surface area was maintained throughout the simulation run since the chitosan–PC complex provided several strong, compact hydrogen bonds at the phosphate polar head. This type of packing allowed the drug–PC complex to acquire an inverted-cone structure with maintained micellar configurations (Ruths et al., 2013) (Figure 8).

### 3.5 *In vitro* antifungal assessment

*Candida albicans ATCC 60193* was used as the test organism to assess the antifungal activity of FTN suspension *in vitro*. The FTN suspension’s MIC was 500 μg/mL.

### 3.6 *In vivo* urinary bladder infection model

**FIGURE 9:**
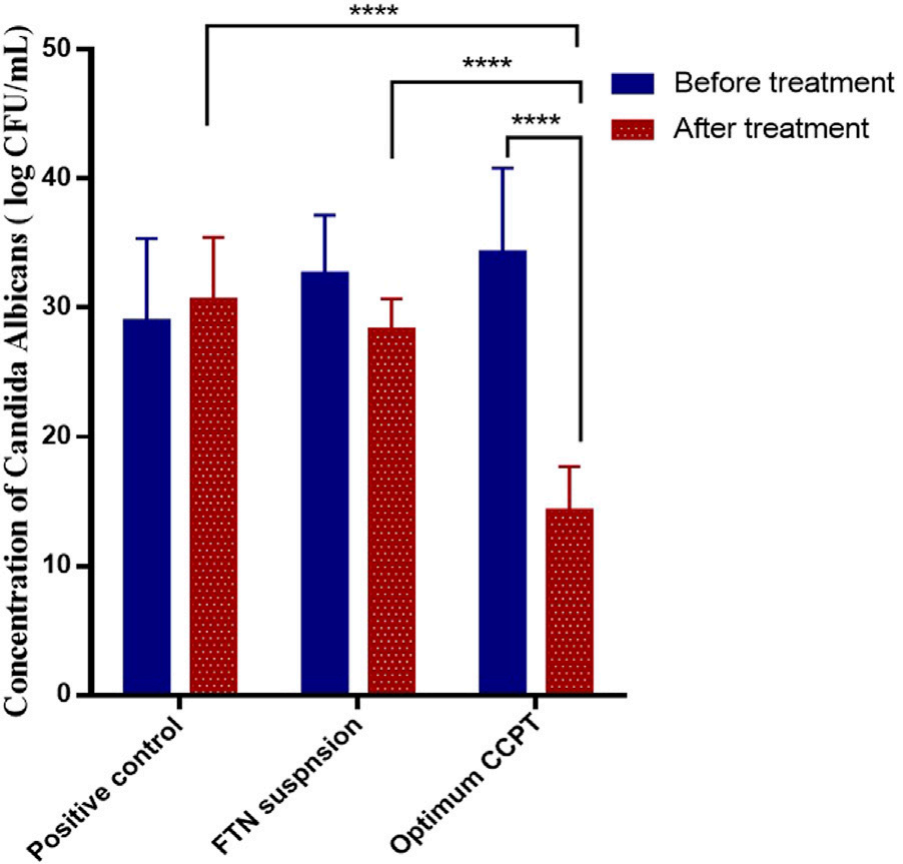
*In vivo* study of positive control, FTN suspension, and FTN–CCPT treated groups.

The antifungal pharmacological effect of FTN was evaluated using a rat model of *C. albicans* urinary bladder infection. Three groups of Wistar rats were intravesically inoculated with *C. albicans* ATCC 10231 suspension to induce infection. As shown in Figure 9, the optimum CCPT formulation demonstrated a marked reduction in fungal burden compared to both the positive control and the FTN suspension groups after 72 h of treatment (*p* < 0.0001). The fungal count in the optimum CCPT group decreased from 34.33 ± 6.49 CFU to 14.33 ± 3.36 CFU, whereas the positive control and FTN groups showed fungal counts of 30.67 ± 4.79 CFU and 28.33 ± 2.36 CFU, respectively, at 72 h post-treatment. These results indicate that the optimum CCPTs achieved a substantial reduction in fungal load compared with both the positive control and the FTN suspension groups. Notably, the optimum CCPT formulation was the only treatment that showed a statistically significant difference associated with the positive control (*p* < 0.0001), while the FTN suspension exhibited no significant effect. Moreover, the CCPT group also confirmed a significant decrease in fungal load compared with the FTN suspension (*p* < 0.0001). The superior antifungal effect of the optimum CCPT system highlights its enhanced ability to deliver the drug to the infection site and effectively suppress fungal colonization within the bladder.

### 3.7 Histopathological examination

**FIGURE 10:**
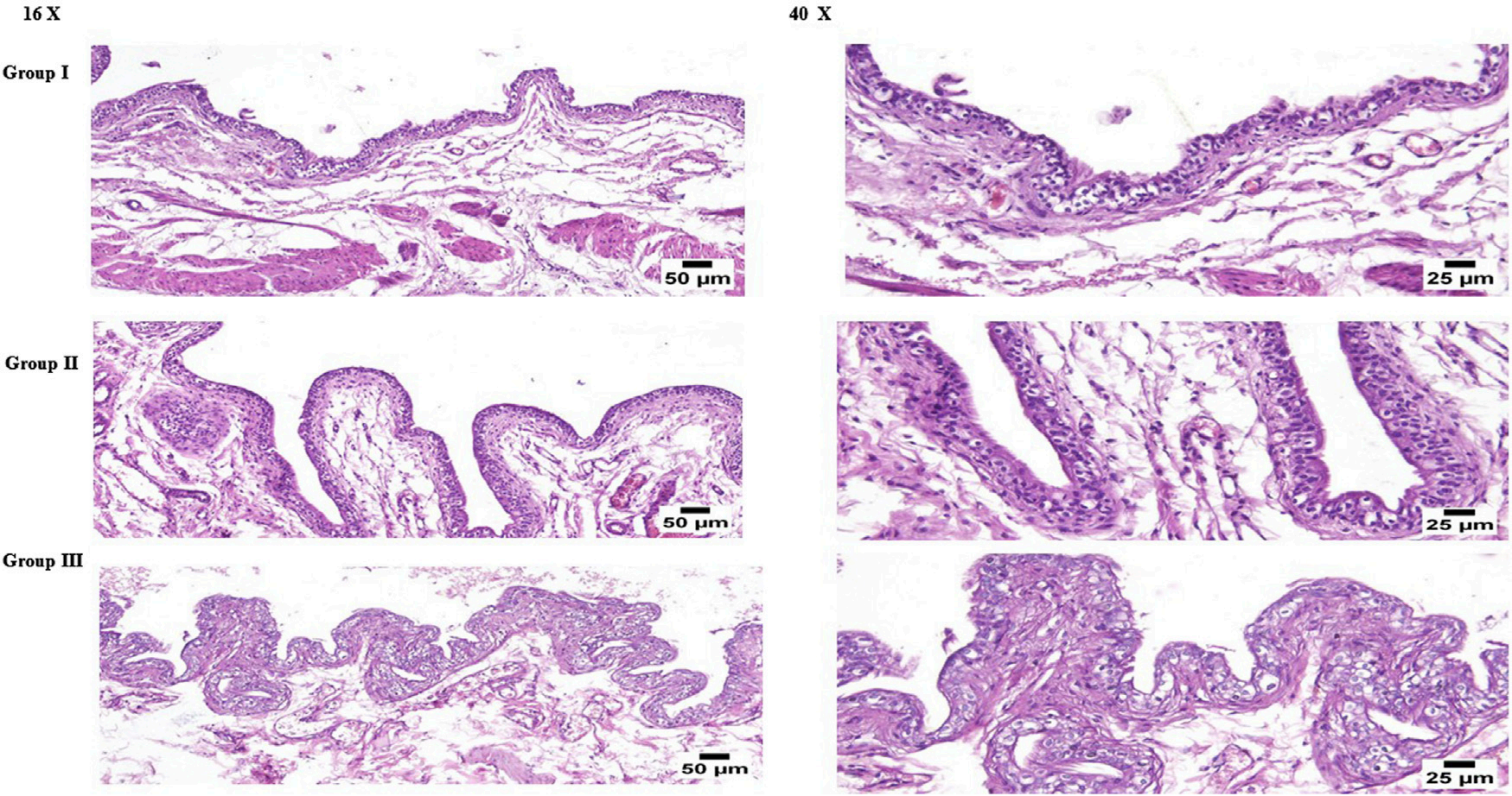
Histopathological sections of the normal untreated rat urinary bladder (group I), urinary bladder of the rat treated with FTN suspension (group II), and the rat urinary bladder treated with optimum CCPTs.

Microscopic examination of the urinary bladder from normal control, FTN suspension, and optimum CCPT groups revealed normal histological architecture, including the mucosa, submucosa, smooth muscle layers, and adventitia (Figure 10). The mucosal lining consisted of transitional epithelium. The muscularis is composed of an outer and inner longitudinal layer and a thick middle circular layer. The adventitia covered the bladder and was composed of loose connective tissue. Mildly congested blood vessels were observed in the submucosa of the FTN suspension group. These results anticipated that optimum CCPTs might have good safety as there was no confirmation of intravesical intolerance found in this estimation; therefore, it might cause no bladder inflammation in clinical trials.

## 4 Conclusion

The current work used a 3^1^.2^1^ full factorial design to successfully fabricate fenticonazole nitrate-loaded terpesomes. The selected terpesomes were further optimized using chitosan as a coat and Gelucire 44/14 as a PEGylated surfactant to increase medication residence duration in the bladder and provide more effective therapy. The optimum chitosan-coated PEGylated terpesomes exhibited small particle size, spherical shape, good entrapment efficiency, and remarkable mucoadhesion. The *in silico* study findings implied the possible good stability upon combining fenticonazole with the formulation constituents. *In vivo* studies showed a significant prevention effect from the optimum chitosan-coated PEGylated terpesomes compared to the fenticonazole suspension. Furthermore, histological examinations demonstrated that the optimum chitosan-coated PEGylated terpesomes were safe. Accordingly, it could be concluded that our developed formulation could be effective for the treatment of bladder disorders via intravesical delivery. Further studies are necessary to prove the therapeutic action of fenticonazole nitrate-loaded terpesomes in humans.

## Data Availability

The original contributions presented in the study are included in the article/Supplementary Material, further inquiries can be directed to the corresponding author.
