# Supplementary material for: Harnessing natural terpenes via PEGylated mucoadhesive vesicles for improved urinary bladder delivery: from in vitro evaluation to in vivo assessment
Source: Front Pharmacol. 2025 Dec 18;16:1685423. doi: 10.3389/fphar.2025.1685423 (PMC12756453; doi:10.3389/fphar.2025.1685423)
Supplement: Supplementary file 1 [file Supplementaryfile1.docx]

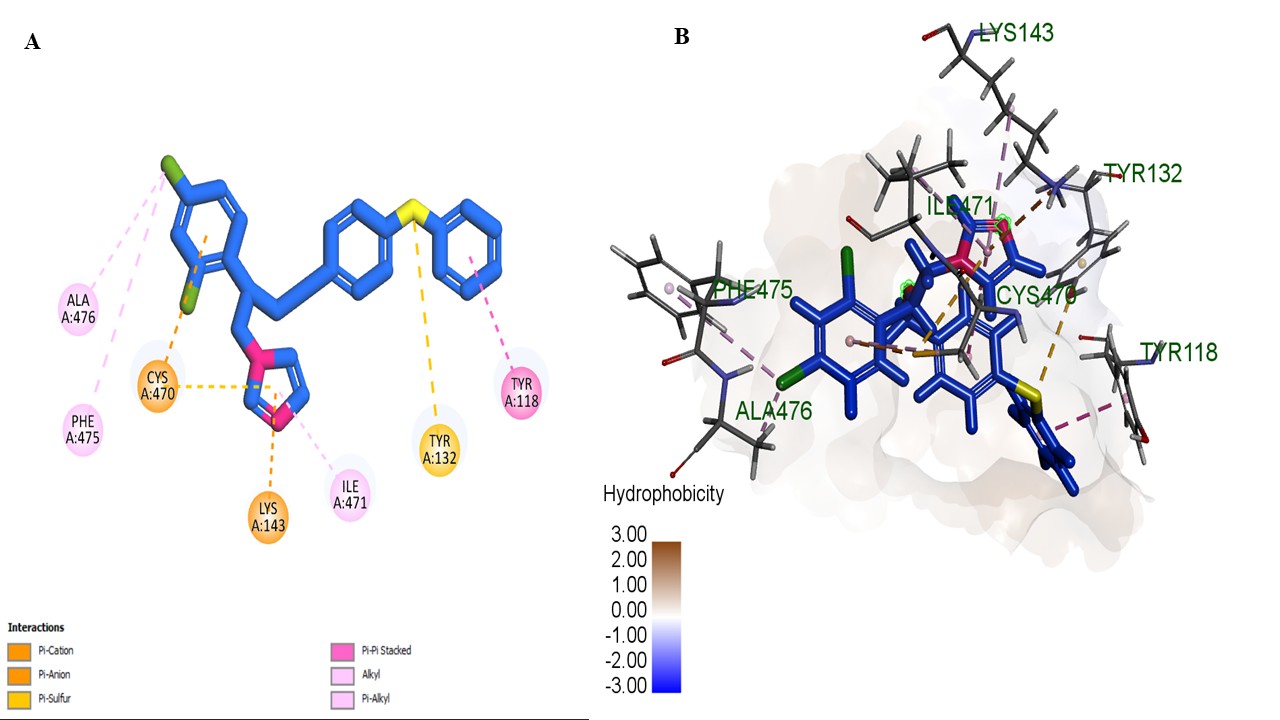


Supplementary Figure 1: 2D and 3D interaction of fenticonazole with the binding site of CYP51.

Supplementary Table 1: The effect of different coating concentrations.

| Chitosan concentration (v/v) | EE% | PS (nm) | PDI | ZP (mV) |
| --- | --- | --- | --- | --- |
| 0.1 | 79.59±0.89 | 218.46±10.44 | 0.52±0.03 | 11.33±5.05 |
| 0.3 | 84.49±2.47 | 227.06±4.73 | 0.63±0.07 | 32.65±2.29 |
| 0.6 | 90.89±1.29 | 1200.86±26.49 | 0.98±0.03 | 34.26±0.63 |
